# Long Non-Coding RNAs in Endometrial Carcinoma

**DOI:** 10.3390/ijms161125962

**Published:** 2015-11-04

**Authors:** Maria A. Smolle, Marc D. Bullock, Hui Ling, Martin Pichler, Johannes Haybaeck

**Affiliations:** 1 Institute of Pathology, Medical University of Graz, Auenbruggerplatz 25, Graz A-8036, Austria; maria.smolle@stud.medunigraz.at; 2 Cancer Sciences, Faculty of Medicine, University of Southampton, Southampton SO171BJ, UK; bullockmd@yahoo.co.uk; 3 Department of Experimental Therapeutics, the University of Texas MD Anderson Cancer Center, Houston, TX 77030, USA; hling@mdanderson.org; 4 Division of Clinical Oncology, Department of Medicine, Medical University of Graz, Auenbruggerplatz 15, Graz A-8036, Austria; martin.pichler@medunigraz.at

**Keywords:** long non-coding RNAs, endometrial carcinoma, cancer

## Abstract

Endometrial carcinoma (EC), the second most common form of gynaecological malignancy, can be divided into two distinct sub-types: Type I tumours arise from hyperplastic endometrium and typically effect women around the time of menopause, whereas type II tumours arise in postmenopausal women from atrophic endometrium. Long non-coding RNAs (lncRNAs) are a novel class of non-protein coding molecules that have recently been implicated in the pathogenesis of many types of cancer including gynaecological tumours. Although they play critical physiological roles in cellular metabolism, their expression and function are deregulated in EC compared with paired normal tissue, indicating that they may also participate in tumour initiation and progression. For instance, the lncRNA MALAT-1 is down-regulated in EC samples compared to normal or hyperplastic endometrium, whereas the lncRNA OVAL is down-regulated in type II disease but up-regulated in type I disease. Other notatble lncRNAs such as HOTAIR, H19 and SRA become up-regulated with increasing EC tumour grade and other features associated with poor prognosis. In the current review, we will examine the growing body of evidence linking deregulated lncRNAs with specific biological functions of tumour cells in EC, we will highlight associations between lncRNAs and the molecular pathways implicated in EC tumourigenesis and we will identify critical knowledge gaps that remain to be addressed.

## 1. Introduction

### 1.1. Endometrial Carcinoma

Endometrial carcinoma (EC) is the second most common form of gynaecologic malignancy, with over 189,000 new cases and about 45,000 deaths worldwide per annum [1,2]. It is estimated that 54,870 new cases will be diagnosed in 2015 in the US with about 10,170 deaths [3].

In general, two main types of EC can be distinguished based on pathological and demographic parameters:

#### 1.1.1. Type 1 EC: Endometrioid Endometrial Carcinoma

Type I EC, also known as endometriod endometrial carcinoma (EEC), is associated with elevated circulating oestrogen levels and emerges from hyperplastic endometrial tissue. In contrast to Type II EC which occurs exclusively in the post-menopausal period, Type I tumours affect women both before and after the onset of menopause. As such, the mean age of patients with Type I EC is slighly lower (40–50 years) than for type II disease (60 years). Furthermore, Type I tumours are typically well differentiated and have a good prognosis, with a low rate of recurrence of approximately 20% [4].

On the molecular level, up to 80% of type I tumours are linked to down-regulation or inactivating mutations of *PTEN* [5,6], which leads in turn to enhanced activity within the PI3K/Akt/mTOR signaling pathway. In addition, constituative activity of the K-ras oncogene or fibroblast growth factor receptor (FGFR) leads to high levels of functioning MAPK, causing increased phosphorylation of the estrogen receptor (ER) in a beta-catenin dependent manner [7]. As a consequence, the downstream transcriptional effects of ER activation promote deregulated cellular proliferation and other activities essential for tumourigenesis [8].

#### 1.1.2. Type II EC: Non-Endometrioid Endometrial Carcinoma

The pathogenesis of Type II EC, termed non-endometrioid endometrial carcinoma (NEEC) is unrelated to circulating oestogen levels. These tumours arise from atrophic endometrial tissue in the post-menopausal state [9]. Type II EC encompases a heterogenous group of tumours which may display histological features consistant with serous, endometrioid or clear-cell differentiation as well as undifferentiated tumour states. As they are typically diagnosed at an advanced stage when distant metastases are already present, the prognosis associated with Type II EC is poor, even with current treatment approaches [10]. Furthermore, approximately half of type II tumours recur within five years after surgical resection [4].

At the level of proteins, NEEC tissue samples show high expression of *TP53* and *P16*, indicating non-functional proteins [11].

### 1.2. Therapeutic Modalities

First-line therapy with curative intention for EC is surgery, which includes removal of the ovaries, fallopian tubes, uterus and cervix, with para-aortic and pelvic lymphadenectomy. Adjuvant therapy, consisting of chemotherapy, hormonal therapy or radiothereapy (RT) may be considered depending on the patients co-morbid state, the completeness of surgical resection margins and the hormone receptor/mutational profile of the tumour. [7]. Chemotherapy followed by radiotherapy and further chemotherapy—the so-called “sandwich” method—is associated with improved overall-survival in patients with advanced disease [12].

## 2. Long Non-Coding RNAs (LncRNAs)

Approximately 2% of the human genome encodes protein, and this means the vast majority of the human genome is not translated into proteins. As this genetic material was thought to be functionless it was initially termed “genomic dark matter” [13]. However over time, multiple information-carrying genes have emerged with important biological and regulatory functions despite remaining untranslated. These include miRNAs (micro RNA), tRNAs (transfer RNA), rRNAs (ribosomal RNA), asRNA (antisense RNA) and snRNA (small nuclear RNA). Most recently, an additional group of non-coding RNAs have been discovered called long-non coding RNAs (lncRNAs), so called because they are longer than 200 nucleotides (nts) in length, in contrast to other classes of non-coding RNA which are generally shorter [14,15]. Although lncRNAs are less conserved than protein-coding genes, considerable homology exists between mammals [16]. They use four main different mechanisms to exert function, the so-called “archetypes”: signalling, decoying, scaffolding and guidance [17].

### 2.1. Signalling LncRNA

LncRNAs are transcribed in a highly regulated manner, either from their own promotor sequence or as a by-product of other transcriptional processes [17]. In turn, lncRNAs regulate the transcription of genes either by interacting with DNA directly or by cooperating with other regulatory elements.

The ability to act immediately without requiring time and energy consuming steps involved in protein translation is perhaps one reason why cells have evolved this form of regulatory mechanism.

“Signalling” lncRNAs include Kcnq1ot1, Xist, Airn, HOTAIR, HOTTIP, linc-p21, PANDA, COOLAIR and COLDAIR. The imprinting genes Airn and Kcnq1ot1, for example, suppress various genes by promoting the repressive functions of histones on chromatin [18]. LncRNAs with signalling functions in particular might be useful as markers for activity of biological processes and disease [17].

### 2.2. Decoy LncRNAs

LncRNAs can bind to and negatively regulate protein effector molecules such as chromatin modifiers and another regulatory factors [19]. Decoy lncRNAs usually exert negative regulation by binding and sequestering protein targets [17]. They are typically called “competing endogenous RNAs”.

DHFR minor (dihydrofolate-reductase) is one example of a decoy lncRNA, which supresses the general transcription factor IIB (TFIIB) and inhibits assembly of other non-coding RNA-DNA complexes. Consequently, knockdown of DHFR minor leads to high levels of TFIIB expression at the major promotor region [20].

### 2.3. Scaffold LncRNA

This archetype represents perhaps the most complex regulatory mechanism. LncRNAs contain various binding domains and as a result, they can bind both repressor and effector molecules simultaneously. Representative lncRNAs include TERC (telomerase RNA), HOTAIR and ANRIL (antisense non-coding RNA).

ANRIL for example interacts with PRC1 and PRC2 (polycomb complexes), forming structures which alter the transcriptional functions of their target *INK4b*, a tumour suppressor [21]. HOTAIR achieves a similar effect by forming a complex with PRC2 [22]. These long intergenic ncRNAs (lincRNAs) use a chromatin signature of actively transcribed genes and are highly conserved [23]. As so-called “flexible scaffolds”, lincRNAs bring together different protein complexes into larger functional units [24]. LincRNA genes are often unevenly conserved, with highly conserved patches that may constitute regions where interaction with protein complexes take place [24].

### 2.4. Guide LncRNAs

As guides, lncRNAs can alter expression of either distantly located genes (*trans*) or neighboring genes (*cis*). They can regulate gene expression in a repressive or activating manner [17,25]. LncRNAs possess specific guiding functions, highlighting that nucleic acids are more than just carriers of the genetic information [26]. For instance, the lncRNA Xist guides many proteins to a large genomic region of the entire X chromosome [27].

LncRNAs guiding in cis are Air, COLDAIR, CCND1, HOTTIP, Xist whereas rDNA, HOTAIR, linc-p21 and Jpx regulate gene expression in *trans* [28].

## 3. LncRNAs and Endometrial Carcinoma

**Figure 1:**
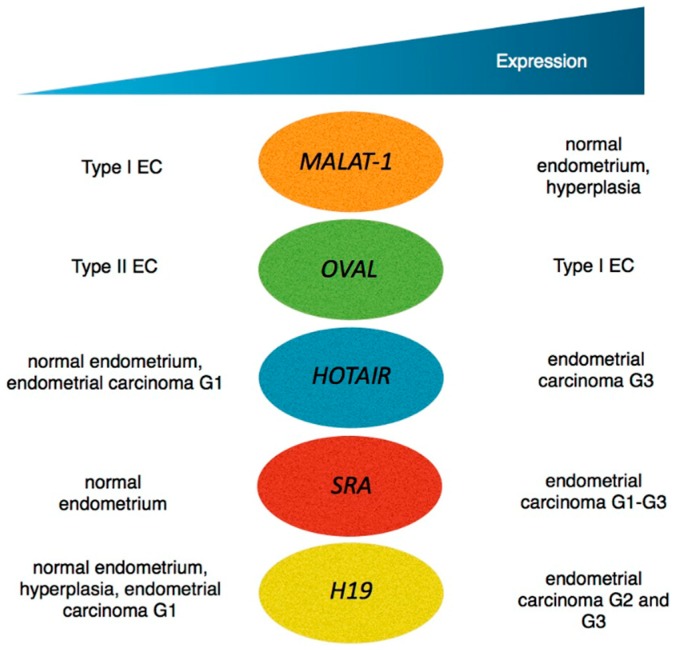
Expression pattern of lncRNAs in different stages of endometrial carcinoma and in comparison to normal endometrium. Type I EC = endometrioid endometrial carcinoma; Type II EC = non-endometrioid endometrial carcinoma. Cases on the left side have lower expression levels as compared with cases on the right side.

In recent years the importance of lncRNAs in both physiology and disease has become increasingly well understood, as in cardiovascular and neurodegenerative diseases as well as in cancers [29,30]. In the following section, we will examine lncRNAs, which have been implicated specifically in the pathogenesis of EC. As a number of lncRNAs are differentially expressed in normal, hyperplastic and dysplastic endometrium (Figure 1) their potential as biomarkers will also be discussed.

### 3.1. MALAT1

MALAT1 (metastasis-associated lung adenocarcinoma transcript 1), has a length of about 8000 nt and is one of the most heavily investigated lncRNAs [31,32,33]. In humans, there are 17 different splice variants of MALAT1. It is upregulated in many different tumours and seems to be involved in tumourigenesis.

The expression of *MALAT1* is induced by the Wnt/beta-catenin signaling pathway. This pathway is commonly abnormally activated in Type-1 endometrial cancer [34]. The wnt/beta-catenin pathway induces transcription of *MALAT1* by interaction of TCF4 with the binding site of the *MALAT1* promoter region [34].

*Procadherin 10* (*PCDH10*) is a tumour suppressor protein that is involved in a variety of malignancies, such as hepatocellular, colorectal, cervical, nasopharyngeal and bladder cancer [35,36,37,38]. *PCDH10* suppresses the transcription of *MALAT-1* by inhibiting the Wnt/beta-catenin pathway. Loss of *PCDH10* leads to strong activation of the Wnt/beta-catenin pathway causing aberrantly high expression levels of MALAT1 [34].

According to Zhau *et al.*, high levels of *MALAT1* can be found in endometrial hyperplasia and low-grade endometrial carcinoma. However, *MALAT1* levels are significantly lower in high-grade endometrial cancer such as clear cell carcinoma and serous papillary carcinoma, as well as metastatic disease [34].

### 3.2. OVAL

The *ovarian adenocarcinoma amplified lncRNA (OVAL)* has three exons and is 1489 nucleotides in size. It is located at the AXI region between the *ACBD6* and *XPR1* genes, a region which lacks protein-coding genes [39]. Initially *OVAL* was thought to encode for various expressed sequence tags (ESTs) and mRNAs, but recent studies have revealed that an alternative first exon isoform, results in the transcription of the lncRNA OVAL [40].

OVAL seems to be an independent target of somatic gene amplification, as co-expression of neighbouring coding genes is not present. Additionally, amplification of *OVAL* does not alter the expression of adjacent genes (*ACBD6, XPR1*). OVAL is unlikely to regulate genes in *cis*, as it is located predominantly in the cytoplasm [39].

OVAL is overexpressed in serous ovarian carcinoma as well as in Type I EC. In contrast, Type II are four times less likely to carry focal upregulation of the *OVAL* gene as compared with its amplification in EEC [39]. p53-regulated genes are upregulated in tissue samples with high expression of OVAL [39]. Among other genes, p53 suppresses the expression of the *IGF-1R* gene by interacting with the Sp1 transactivator protein [41,42].

### 3.3. HOTAIR

The HOX transcript antisense intergenic RNA (HOTAIR) is located at the antisense strand of the *HOXC* gene cluster on chromosome 12. Overexpression of HOTAIR is associated with poor prognosis in various malignancies, such as hepatocellular, colorectal, breast and nasopharyngeal carcinoma [43,44,45,46].

As mentioned above, estrogen is involved in the carcinogensis of type I EC. According to Bhan *et al.*, *HOTAIR* constitutes an estradiol-responsive gene; the expression of HOTAIR is induced by estradiol, estrogen receptors and general transcription factors of RNA polymerase II [47]. Thus, HOTAIR is not only aberrantly expressed in breast cancer but also in endometrial carcinoma [28]. According to He *et al.*, HOTAIR was upregulated in nearly three quarters of endometrial cancer samples (63 out of 87), whereas only 20% of normal endometrium were positive for this specific lncRNA (4 out of 30). More dedifferentiated tumour samples (G3) showed higher expression levels than G1 tumour samples. Additionally, in hyperplastic endometrium, gene up-regulation could be detected in about 50% of cases (5 out of 12) [48].

HOTAIR is also associated with metastatic spread, as high levels of HOTAIR expression correlates positively with metastases and decreased overall-survival in EC [48]. In a xenograft EC model HEC-1A cells treated with HOTAIR siRNA lentivirus were implanted into mice. The resulting depletion of HOTAIR expression *in vivo* significantly suppressed endometrial tumourigenesis and led to smaller tumour sizes [49,50].

### 3.4. SRA

The Steroid receptor RNA activator (SRA) plays a critical role in the regulation of eukaryotic gene expression induced by steroid receptors [51]. SRA is upregulated in breast cancer as well as in other steroid-responsive tumour tissues, such as ovarian carcinoma [52].

In endometrial adenocarcinoma, consistently high levels of SRA expression are found regardless of histological tumour grade. However, in healthy reference tissue samples, SRA is expressed at very low levels implying an early role in tumourigenesis [52]. Interestingly, SRA-transgenic mice in a study by Lanz *et al.* did not develop any tumours despite overexpression of SRA [52]. Further experiments revealed increased apoptosis rate in SRA-transgenic mice counteracting the raised mitotic activity. A specific protein interacts with p53 and NFκβ-pathways, but also binds to the RNA substructure STR1 of SRA. This protein might explain the involvement of SRA in apoptotic and inflammatory processes [53]. Therefore, it is proposed that *SRA* upregulation in tumour tissue constitutes a reactive process in order to decelerate increased proliferation of tumour cells [52].

### 3.5. H19

The lncRNA H19—together with insulin-like growth factor 2 (IGF2)—is important during the normal menstrual cycle and in early pregnancy, as it decreases cellular stress caused by serum starvation [54]. Estradiol (E2) positively influences the up-reguation of H19 in the endometrium whereas progesterone leads to down-regulated H19 expression [55].

Furthermore, H19 can increase the expression of genes that are important for tumourigenesis. For example, members of the mitogen-acitvated protein kinase superfamily including c-jun, JNK1/2 and the extracellular signal-regulated kinase 1 and 2 are induced by H19. These genes are important for migration of tumour cells [56,57,58].

Moreover, H19 suppresses the expression of the beta-5, beta-3 and alpha-4 integrins that are required for cell to cell adhesion. Therefore, downregulation of these integrins may lead to raised motility and increased invasive potential of tumour cells [59].

In normal endometrial epithelium, H19 is expressed at a low level, but the expression-levels increase in hyperplastic endometrium. In endometrial carcinoma, H19 levels are very high and increase still further with ongoing dedifferentiation of the tumour tissue. On the contrary, healthy uterine stroma-cells present with higher levels of H19 as compared with stromal tumours [60]. This is contradictory to the fact that H19 acts as a sponge to bind the microRNA let-7, as this microRNA usually inhibits *Igf1r*. Inhibition of *Igf1r* leads to a decrease of IGF1R protein. High levels of IGF1R are necessary in order to allow proliferation of the endometrial stroma. During the menstrual cycle, raised H19 levels cause sequestration of let-7, increased IGF1R levels and therefore endometrial stromal hyperplasia [61]. In stromal tumours, alternative pathways seem to be used to allow tumour growth.

## 4. Conclusions

Altered levels of sex hormones are involved in the development of EC. The lncRNAs SRA, H19 and HOTAIR are directly affected by changes in sex hormone-levels and may play a role in the carcinogenesis of EC. E2 seems to constitute an important linkage between expression of these lncRNAs and altered levels of sex hormones as it positively correlates with up-regulation of *H19* and *HOTAIR* in endometrial carcinoma [47,55].

Overall, lncRNAs show specific and altered expression patterns in endometrial carcinoma compared to normal endometrial tissue (see Figure 1). In the absence of definitive evidence regarding the role of lncRNAs in endometrial cancer, models have emerged which show lncRNAs can be targeted in order to archieve anti-tumourigenic effects. Small interfering RNAs (siRNAs), antisense oligonucleotides (ASOs), ribozymes and aptamers are used to effectively modulate lncRNA expression [62]. The most often applied method to inhibit lncRNAs is the use of siRNAs. Constituting short stretched, double-stranded RNAs, siRNAs effectively target RNA molecules. They form the RNA-induced silencing complex (RISC) and induce post-transcriptional silencing of RNA targets [63]. MALAT1 can be depleted by siRNAs in hepatocellular carcinoma, leading to cell-cycle arrest and therefore decreased cell proliferation [64].

Further investigation is needed in order to discover the exact functions of specific lncRNAs in tumourigenesis. They may constitute therapeutic targets and prognostic or predictive biomarkers that would help to improve tumour therapy.
